# EEG Asymmetry and ERN: Behavioral Outcomes in Preschoolers

**DOI:** 10.1371/journal.pone.0155713

**Published:** 2016-05-25

**Authors:** J. Patrick Begnoche, Rebecca J. Brooker, Matthew Vess

**Affiliations:** Department of Psychology, Montana State University, Bozeman, Montana, United States of America; University of California, San Francisco, UNITED STATES

## Abstract

Research has documented reciprocal influences between approach-related and inhibition-related neural activity in adults. However, associations between neural systems of approach and inhibition have not been tested in children. It is thus unclear whether these links are present early in life and whether associations between neural systems of approach and inhibition have long-term behavioral consequences. To address these gaps in the literature, we used electroencephalography (EEG) to examine associations between approach-related neural activity (i.e., hemispheric asymmetry) and inhibition-related neural activity (i.e., error-related negativity [ERN]) in preschool-aged children. Furthermore, we explored whether interactions between asymmetry and ERN predicted social inhibition, a precursor to anxiety problems, or symptoms of Attention Deficit Hyperactivity Disorder (ADHD) six months later. Similar to research on adults, greater left asymmetry (i.e., greater approach-related neural activity) was correlated with reduced ERN amplitude (i.e., weaker inhibition-related neural activity). The interactive effect of asymmetry and ERN amplitude did not predict ADHD symptoms, but did predict social inhibition. When ERN was greater, less left asymmetry was associated with higher levels of social inhibition. Results were most prominent at parietal EEG sites. Implications for understanding the development of the overlap in neural systems of approach and inhibition are discussed.

## Introduction

Systems of trait-level motivation play a crucial role in the genesis of both goal-oriented behaviors and symptoms of psychopathology. For instance, both children and adults show positive associations between propensities for inhibition, or engaging in avoidant behavior, and internalizing symptoms [1–3]. Likewise, positive, although less robust, associations between propensities for activation, or approach, and levels of externalizing symptoms exist [4], [5]. In adults, greater tendencies for approach-related behaviors are associated with dampened propensities to inhibit one’s behavior [6], [7]. Evidence for this type of antagonistic relation between motivational systems early in life, however, is lacking. Given the importance of motivational systems for psychological health, understanding whether this interaction between approach- and inhibition-related systems is present in young children could help elucidate the development of mechanisms underlying risk for psychological disorder. In the current study, we tested for initial evidence of associations between approach and inhibition systems early in life and their longitudinal interactive effects on early internalizing and ADHD symptoms.

### BIS, BAS, and the Joint Subsystems Hypothesis

Distinct systems of inhibition and approach were first proposed as part of Reinforcement Sensitivity Theory [8]. This theory posits two general motivational systems: the *Behavioral Inhibition System* (BIS) and the *Behavioral Activation System* (BAS). The BIS is a neurobiological system activated by goal conflict that catalyzes avoidance of goal pursuit, initiates fear and anxiety, and enhances individual sensitivity to punishment and novelty [8]. In contrast, the BAS is a neurobiological system that is activated by goal pursuit and initiates approach behaviors, enhances insensitivity to punishment, and increases sensitivity to reward and novelty [8]. Stable individual differences in both BIS and BAS sensitivity, or propensities for BIS and BAS to be activated, exist and are predictive of psychological outcomes. BIS activity is negatively associated with positive affect and overall well-being [9], and positively associated with psychological problems for which symptoms of distress are largely expressed in an inward fashion (i.e., internalizing problems), including depression, anxiety, negative affect, and social avoidance [2], [4]. On the other hand, although greater BAS sensitivity is generally linked to positive psychological outcomes [10], extreme BAS sensitivity may be problematic. For example, BAS activity has been positively linked to psychological problems for which distress is overt, such as ADHD symptoms [11] and conduct problems [4]. Findings such as these provide evidence that individual differences in BIS and BAS sensitivity predict distinct behavioral and emotional outcomes and thus support Reinforcement Sensitivity Theory’s postulate that the BIS and BAS are distinct motivational systems.

Nonetheless, a recently proposed *Joint Subsystems Hypothesis* posits that the BIS and BAS jointly function to influence behavior [6]. Specifically, greater BAS activation is believed to antagonize BIS activity in order to sustain active goal pursuit, resulting in an interactive effect on behavior. Recent research [7] utilized psychophysiological indicators of BIS and BAS activity to provide support for this hypothesis. The psychophysiological approach of this work was critical; it allowed for the separate quantification of both BIS and BAS at the level of neural systems while minimizing interference from more distal behavioral systems (e.g., effortful regulatory systems that may suppress observable behaviors). BIS activity was indexed as the amplitude of the Error-Related Negativity (ERN), a neural marker associated with cognitive conflict and error commission ([12], [13]), however see review [14] for alternative interpretations). The ERN has been linked to self-reported BIS [15] and to the BIS-like traits of sensitivity to aversive events [16], anxiety [9], [17], and punishment [18]. BAS activity was indexed as the relative activity in the brain’s left versus right frontal hemispheres. Greater activity in the left hemisphere, or left frontal asymmetry, is associated with self-reported BAS and with the BAS-like traits of approach and reward sensitivity [19–21]. Consistent with the Joint Subsystems Hypothesis, Nash and colleagues [7] found that greater left frontal asymmetry was correlated with smaller ERN amplitudes, as would be expected if BAS activity functioned as an antagonist for BIS activity.

### BIS, BAS, and Early Development

To date, empirical studies of the Joint Subsystems Hypothesis [6], [7] have focused exclusively on adults. Research on child temperament, defined as constitutionally based individual differences in activity, reactivity, and emotionality [22], however, suggests that behavioral correlates of the BIS and BAS are present much earlier in life. In addition, both the neural markers of BIS (ERN) and BAS (hemispheric asymmetry) utilized in previous tests of the Joint Subsystems Hypothesis can be reliably measured in children. The ERN has now been elicited in children as young as 3 years of age [23] and has been associated with temperamental fearfulness as early as age 4 [24]. Likewise, hemispheric asymmetry can be reliably measured as early as 3 months of age [25–27] and is associated with temperament traits that map onto adult conceptualizations of BAS, including greater positive affect, approach, and sociability [26]. That these neural markers of BIS and BAS can be detected in young children raises questions about whether the joint influences predicted by the Joint Subsystem Hypothesis are evident early in life and whether interactions between BIS and BAS can be utilized to understand longitudinal outcomes linked to psychological dysfunction.

Indeed, interactions between BIS and BAS remain largely unlinked to longitudinal outcomes. In children, the behaviors and neural processes associated with BIS and BAS have been identified as potential risk factors for psychological problems. For example, temperamental fearfulness is the behavioral hallmark of social inhibition, an established risk factor for the development of anxiety problems during childhood and adolescence [28], [29]. Additionally, high temperament-based approach is positively associated with ADHD symptoms [30], [31]. As might be expected as indices of BIS and BAS, respectively, greater ERN is associated with greater risk for social inhibition [32] and anxiety problems [33], while greater left frontal asymmetry is associated with greater impulsivity, approach [34], and enhanced risk for ADHD [35].

To the degree that BAS mutes BIS propensities such as fear and withdrawal, risk for anxiety problems linked to greater ERN may be diminished for children who show greater left frontal asymmetry. Indeed, at the behavioral level, children high in self-reported BIS and low in self-reported BAS displayed significantly more social anxiety compared to children who were high in both BIS and BAS [2]. At the biological level, this would lead to an expectation that children who show greater ERN amplitudes and less left asymmetry would show the greatest levels of social inhibition, denoting a compounded risk for anxiety problems. Similarly, greater left asymmetry coupled with muted ERN may reflect, at the biological level, the greatest level of risk for ADHD problems, as left asymmetry would not offset–but amplify–already-low propensities for withdrawal [36]. As noted, however, such interactive effects have not been tested in children using biological markers of BIS and BAS sensitivity.

The current study had two aims. First, we conducted a novel test of the Joint Subsystems Hypothesis by assessing whether greater ERN was associated with less left frontal asymmetry during preschool. Given evidence for similar behavioral and biological substrates of BIS and BAS during preschool and adulthood, we hypothesized that greater left frontal asymmetry would be associated with diminished BIS activity in young children. In children, however, the frontal lobes are less fully developed relative to adults [37]. This immaturity frequently results in broadly distributed patterns of neural processing [38], [39]. Thus, because measures of parietal asymmetry may provide additional information about motivational tendencies in young children, we expected similar patterns of associations between ERN and both frontal and parietal hemispheric asymmetry.

Our second aim was to test whether ERN activity and hemispheric asymmetry interacted to predict longitudinal outcomes in children. We hypothesized that less left frontal asymmetry combined with greater ERN would predict greater levels of social inhibition. We also hypothesized that greater left frontal asymmetry coupled with reduced ERN would predict greater levels of ADHD. Again, both frontal and parietal measures of asymmetry were used.

## Method

### Participants

This study was approved by The Pennsylvania State University Institutional Review Board (IRB #27683). Written informed consent was obtained from the parent or legal guardian of each child who participated. Children provided verbal assent. Sixty-six families who were part of a larger, ongoing study of temperament in toddlers were invited to participate in the current study. Children were required to be 4.5 years of age and free of neurological disorders, developmental delays, and psycho-stimulant medications at the time of the invitation. All children were typically-developing. At the time of recruitment, one family withdrew from the parent project, 7 families failed to respond to the invitation to participate, 3 families had moved away from the area, 13 families declined to participate, and one family did not show for their laboratory visit and did not reschedule. The sample thus included 41 preschoolers (20 girls; *M* age = 4.59; *SD* = 0.13) who were representative of the area from which they were recruited with respect to socioeconomic status and racial and ethnic diversity. Parents identified the majority of children (87.5%) as Non-Hispanic Caucasian, 5.0% as African-American, 5.0% as Asian American, and 2.5% as of Hispanic ethnicity. Families reported annual incomes ranging from less than $15,000 to over $60,000. The highest proportion of families (47.5%) reported annual incomes of more than $60,000.

### Procedure

All children visited the laboratory at age 4.5 for a psychophysiological assessment. Families were also invited to participate in an age 5 follow-up assessment for which a packet of questionnaires was mailed to parents and then returned to the laboratory.

### Age 4.5 Assessment

Upon arrival to the laboratory, parents verbally reported children’s handedness following consent procedures. Children were fitted with a 128-channel Hydrocel Geodesic Sensor Net (Electrical Geodesics, Inc.) for EEG collection. Children then completed three laboratory episodes: a resting baseline, an age-appropriate computerized task, and a second resting baseline. For each baseline period, children were instructed to alternate between resting for 1 minute with eyes open and 1 minute with eyes closed for a total of 5 minutes. During the eyes open period, children were instructed to fix their gaze upon a slowly-moving shape on the computer screen to help reduce eye movement artifacts. This procedure is similar to those used in previous research [40].

**Fig 1:**
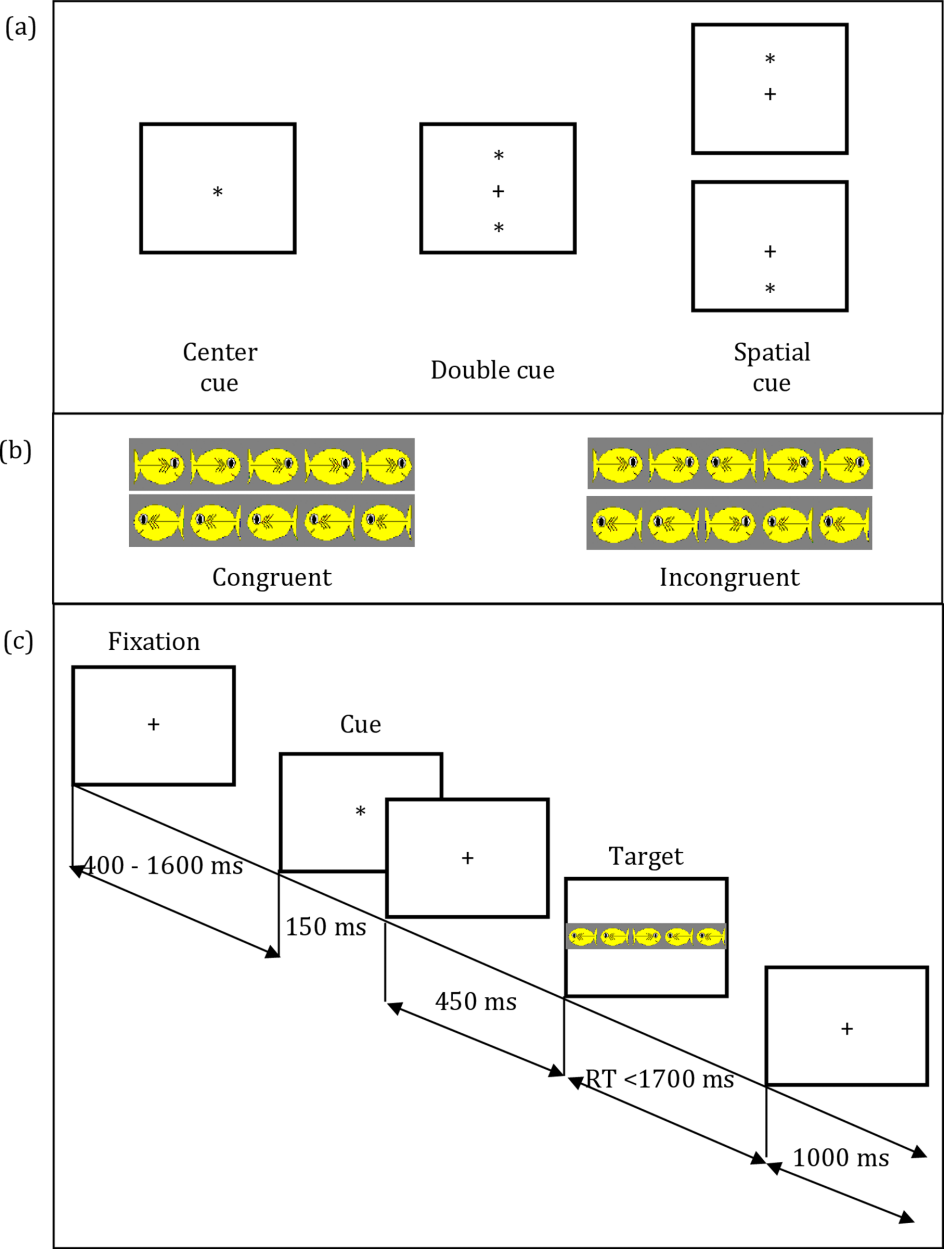
Experimental procedure. (a) The three cue conditions, (b) The four stimuli used in the present study, and (c) an overview of the procedure.

Between baselines, children completed the Attention Network Test (ANT; [41]) on a Dell PC using E-prime 1.1 (Psychology Software Tools, Inc: Pittsburg, PA). An ANT session comprised two experimental blocks of 64 trials each. If necessary, a short break between blocks was permitted. Each trial started with the presentation of a fixation cross for 400 ms. On some trials, a warning cue was then shown for 150 ms and represented one of three conditions: double cue, spatial cue, or no cue. As previously reported [24], differences between cue conditions were nonsignificant and so cue conditions were collapsed. Following a second fixation period of 450 ms, an array of cartoon fish including a target fish and four flanking fish appeared and remained on the screen until either the participants responded by denoting via button press the direction faced by the target fish (right or left) or 1,700 ms had elapsed. Children received feedback on each trial in the form of a smiling or frowning face taken from the NimStim Set of Facial Expressions [42]. The full ANT procedure is outlined in Fig 1. Accuracy and response time were recorded for each trial. To eliminate trials that may have reflected non-deliberate behavior, such as guessing, trials with response times of less than 200 ms were removed from both the behavioral and electrophysiological data [43].

Prior to beginning the task, children viewed printed, paper copies of each type of trial contained in the ANT. Participants were required to point out the target stimulus and indicate which response button they would press to register their answer for that trial. If any trial was answered incorrectly, the experimenter would re-explain the rules of the task and readminister the paper practice trials. Following the paper practice trials, participants completed a series of computerized practice trials to acclimate children to the application of the rules to the computer task. If a large number of errors were made during the practice trials or if, following the completion of the practice trials, the child indicated that s/he was not yet ready to start the task, the computerized practice trials were re-administered. A total of 5 paper and 16 computerized practice trials were initially offered to children. In general, most children completed only these 21 practice trials; however, because practice trials were re-administered as needed, not all children completed the same number of practice trials.

EEG data were acquired during baseline and the ANT using NetStation (version 4.3.1) acquisition software (Electrical Geodesics, Inc.: Eugene OR). Data were sampled at a rate of 500 Hz with a gain of 1,000. Preceding data collection, impedances were reduced to less than 80 kΩ, consistent with the recommendation of the manufacturer for work with children at this age. EEG data were filtered throughout recording using an analog filter. The high-pass filter was set at the manufacturer default of 0.1 Hz. An elliptical IIR filter was used to low-pass filter incoming data at 100 Hz, well above the Nyquist frequency, to prevent aliasing. All channels were referenced to electrode Cz during data collection.

EEG data processing was conducted offline using a semiautomated procedure in Brain Vision Analyzer (BVA; Brain Products: Gilching, Germany). All data were re-referenced to an average of the two mastoid channels. EEG data were then highpass filtered at 0.10 Hz (12dB rolloff). Data from each participant were submitted to an Independent Components Analysis in EEGLab Version 8.0.3b [44] in order to remove eye movement or eye blink artifacts. Using a semiautomated procedure, artifacts were defined when one of the following conditions were met: a step of more than 75μV transpired between data points, a difference of 150 μV occurred within a single segment, an absolute voltage exceeded 200 μV within a single segment, or amplitudes less than 0.5 μV occurred within a 50 ms period. All segments were also visually inspected for any remaining artifacts.

To score alpha asymmetry, EEG data collected during the baseline episodes were collapsed and lowpass filtered at 30 Hz. Segments of 1.024 s were extracted from the continuous EEG data using the entire data segment. Artifact-free data were submitted to a Fast-Fourier Transform using a Hamming window with 50% segment width overlap. Power (μV^2^) was derived for the child alpha (6–10 Hz; [40] frequency band, for frontal (Fp1, Fp2), and parietal (P3, P4) electrode sites. Power values were transformed using a Log10 transformation to correct for nonnormal distribution. Asymmetry scores were calculated for homologous electrode pairs by subtracting alpha power in the left hemisphere from alpha power in the right hemisphere. Because alpha reflects an inverse of activity, positive scores on this metric imply relatively greater left-than-right cortical activation (left asymmetry) while negative scores imply relatively greater right-than-left cortical activation (right asymmetry).

To score ERN, EEG data collected during the ANT were divided into 1600 ms segments starting 600 ms prior to each participant’s response. Segments were baseline corrected by subtracting the average activity from -600 to 0 ms from each data point. Individual averages for artifact-free segments were generated for correct and error trials and were lowpass filtered at 30 Hz. ERN and correct trial negativity (CRN) averages were not created for children who did not commit a minimum of 5 errors or for whom an excessive number of artifacts resulted in less than 5 usable trials [45]. This resulted in an average of 51.61 trials (*SD* = 26.73; *range* = 5–99) for the calculation of CRN and 21.25 trials (*SD* = 19.79; *range* = 5–109) for the calculation of ERN.

To control for individual differences in neural activity on correct trials, we created an ERN difference wave (ΔERN) by subtracting the correct trial average from the error trial average (ΔERN = ERN-CRN). An automated procedure was used to score the peak of the ΔERN as the mean amplitude (+/-20 ms) around the most negative peak of the difference wave occurring in the window of -100 ms to 100 ms following the response. Scored in this fashion, greater ΔERN corresponds to greater negative amplitudes. Consistent with previous work, CRN, ERN and ΔERN were scored at Fz, Cz, and Pz [24], [46], [47].

### Age 5 Assessment

When children reached 5 years of age, parents were asked to complete a packet of questionnaires as part of a larger longitudinal study. The questionnaire packet included the MacArthur Health and Behavior Questionnaire (HBQ; [48]), which assesses dimensional ratings of emotional and behavioral symptomology, physical health, and social adaptation in young children. Given the aims of the current study, we focused on two scales of the HBQ: Social Inhibition and ADHD symptoms. For these, parents were asked to rate, on a 3-point Likert scale, the degree to which certain behaviors are characteristic of their child (0 = Never true, 2 = Often true). The Social Inhibition scale comprises 3 items that ask about the degree to which children are shy or inhibited around unfamiliar people. The ADHD scale comprises 15 items that and ask about the degree to which children evidence impulsive or inattentive behaviors. Reliabilities based on data from the current sample were α = 0.68 and α = 0.90 for the Social Inhibition and ADHD scales, respectively.

### Missing Data

For the current analyses, six left-handed children were excluded from the data set, given evidence that patterns of hemispheric activation my differ in left- and right-handed individuals [49]. In addition to this, 4 children did not provide usable baseline data, either due technical error (*n* = 1), task refusal (*n* = 2) or excessive artifact (*n* = 1); ΔERN could not be scored for 5 children who did not commit a sufficient number of errors [45]; and 5 families did not complete the follow-up measure. This resulted in full data being available for 26 children for tests of associations between BIS and BAS and for 21 children for tests of BIS/BAS association with childhood outcomes. These are comparable to the final sample size (*N* = 26) reported by Nash, Inzlicht, and McGregor’s Study 1 [7].

## Results

### Preliminary ERN analyses

**Fig 2:**
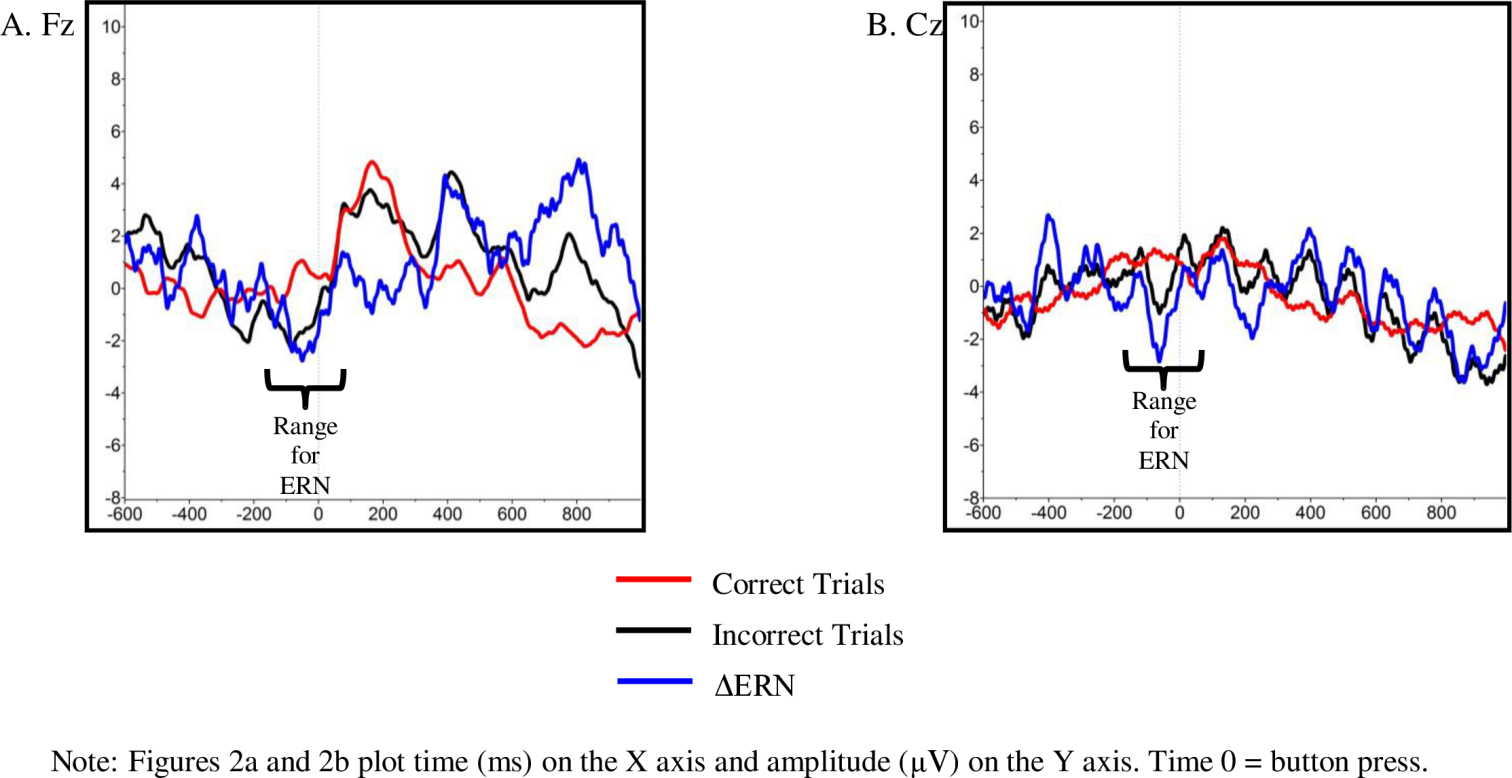
Grand Average Waveform at (A) Fz and (B) Cz.

To maximize available power, the presence of ERN was established in the full sample of children with ERN data (i.e., not excluding left-handed individuals) using a 2 (trial type: correct, error) by 3 (electrode site: Fz, Cz, Pz) repeated measures ANOVA. Greenhouse-Geisser corrections were used to correct for potential violations of sphericity. Consistent with expectations for ERN, amplitudes for incorrect trials were more negative than amplitudes for correct trials (*F*(1, 32) = 9.94, *p*< 0.01). As has been seen in previous work with children, amplitudes for correct and error trials showed trend-level differences across electrode sites (*F*(2, 64) = 2.54, *p*< 0.10). As suggested by Fig 2, a probe of the interaction between electrode site and trial type revealed that a significant ERNs and Fz (*t*(35) = -2.43, *p* < 0.05, *d* = -0.54)) and Cz (*t*(34) = -2.01, *p* < 0.05, *d* = -0.46)), but not at Pz (*t*(33) = -1.24, *p* > 0.10). As previously suggested [24], this pattern of results likely reflect the lack of localization of ERN to a single electrode site by age 4.5 years.

Based on these results, subsequent analyses focused on ERN difference scores (ΔERN; incorrect–correct) at Fz and Cz electrodes. A difference wave approach was selected based on the recommendations of this strategy for researchers who which to isolate neural processes while controlling for concurrent neural activity that is unrelated to the process of interest [50]. That is, by controlling for response-related activity that is also present on correct trials, we were able to look more specifically at error-related negativity.

### Testing the Joint Subsystems Hypothesis in Preschoolers

Children performed well, overall, on the ANT. An average of 76.60% of trials were answered correctly (*SD* = 13.97) in the full sample. Only one child scored at a rate of less than 50% correct (% correct = 48.35%). Results are identical with or without this participant in the analyses and so they are included in results as presented here. Consistent with previous work, the mean reaction time for incorrect and correct trials was 904.52 ms (*SD* = 199.41) and 936.10 ms (*SD* = 141.43), respectively.

Descriptive statistics for study variables are reported in Table 1.

**Table 1:** Descriptive Statistics for Study Variables.

	*N*	Mean	*SD*
ΔERN Fz	36	-15.40	15.78
ΔERN Cz	30	-13.07	14.29
Frontal Asymmetry	31	0.02	0.07
Parietal Asymmetry	31	0.05	0.18
Social Inhibition	29	0.92	0.48
ADHD Symptoms	29	0.68	0.36

**Fig 3:**
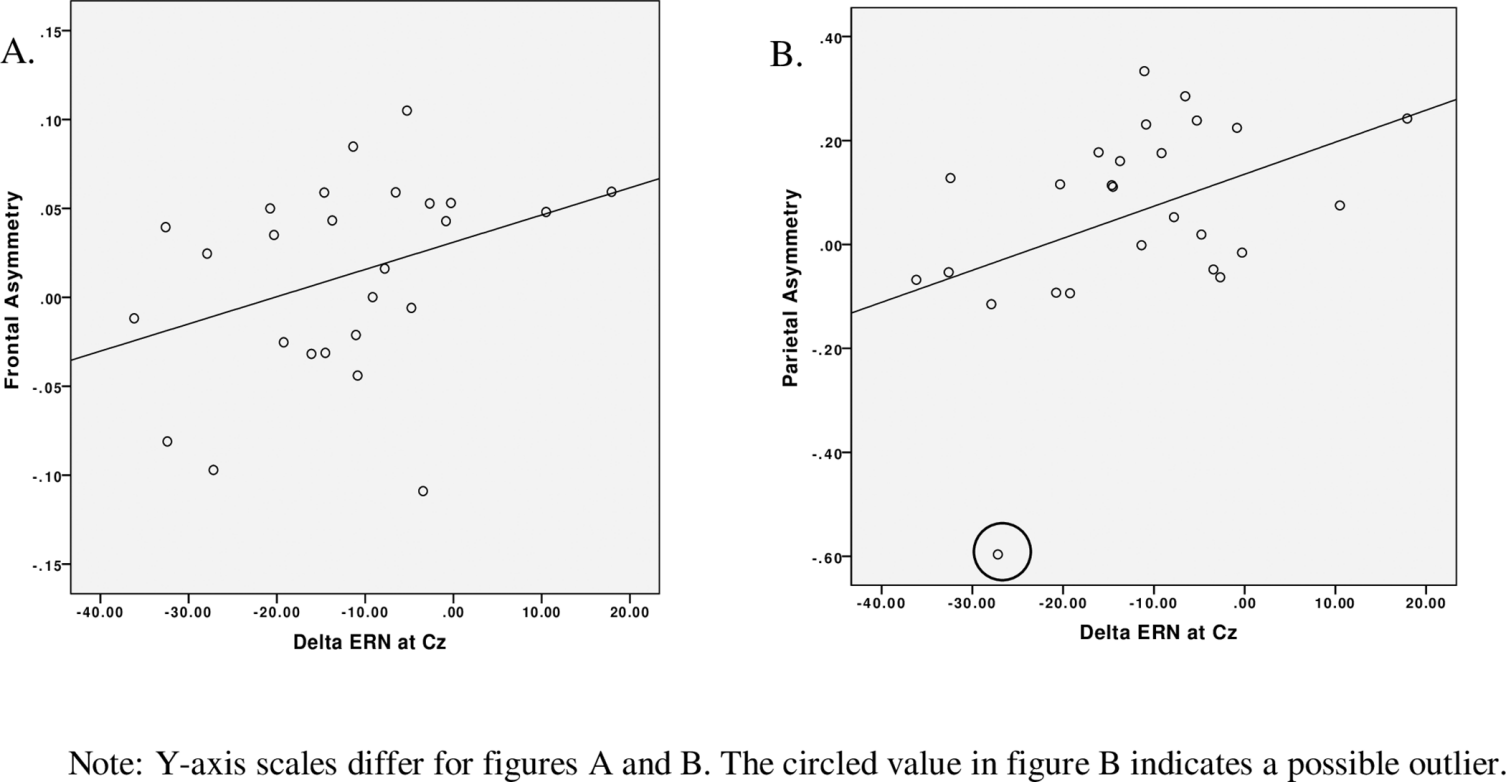
Scatter plot diagrams of the correlations between Cz ΔERN amplitude and (A) frontal and (B) parietal asymmetry.

To replicate the procedures followed by Nash & colleagues, Pearson correlation coefficients were used to assess associations between asymmetry scores and ΔERN amplitudes. At Fz, ΔERN was not correlated with either frontal (*r*(25) = .06, *n* = 27, *p* = .77) or parietal asymmetry (*r*(25) = .14, *n* = 27, *p* = .50). However, as shown in in Fig 3, ΔERN amplitudes at Cz were marginally associated with frontal asymmetry in preschoolers, *r*(24) = .36, *n* = 26, *p* = .07, and were significantly associated with left asymmetry at parietal sites, *r*(24) = .42, *n* = 26, *p* = .03. In both cases, greater left asymmetry was associated with smaller (less negative) ΔERN amplitudes. We note that a possible outlier appears to exist as illustrated in Fig 3B. When this data point is removed, the association between parietal asymmetry and ΔERN is slightly reduced in magnitude, *r*(23) = 0.38, but does not statistically differ from the original correlation (Fisher’s *r* to *z* = 0.16, *p* = 0.44).

Consistent with the suggestions of Allen and colleagues [51], we conducted exploratory multiple regression analyses to examine whether alpha power in right or left electrodes contributed independently to the prediction of ΔERN amplitude at Cz. Power in both right (β = 1.31, *p* = .10) and left (β = -1.44, *p* = .07) frontal electrodes was marginally associated with the ΔERN at Cz, albeit in opposite directions. Greater right prefrontal activity (less alpha power in the right hemisphere) was associated with enhanced (more negative) ΔERN amplitudes while greater left prefrontal activity was associated with reduced ΔERN amplitudes. In contrast, neither power in the right (β = -0.07, *p* = .80) nor left (β = -0.36, *p* = .21) parietal electrode was associated with ΔERN. This suggested that, for parietal measures, the relative difference in alpha power was critical for the link between asymmetry and ΔERN.

### ERN and Alpha Asymmetry as Predictors of Child Outcomes

We next used hierarchical regression models investigated whether ΔERN at Cz and alpha asymmetry interacted to predict Social Inhibition and ADHD symptoms. Analyses focused on ΔERN at Cz given that ΔERN at Fz was not associated with alpha asymmetry. Although Social Inhibition and ADHD symptoms were tested as outcomes in separate models, analytic procedures were identical. Given that the patterns of correlations reported above suggest that both frontal and parietal asymmetry were associated with ΔERN at Cz, we also tested frontal and parietal asymmetry separately for each outcome. For all analyses, the centered main effects for alpha asymmetry and ΔERN at Cz were entered in the first step and the interaction between alpha asymmetry and ΔERN at Cz was entered in the second step. The results of these analyses are presented in Table 2.

**Table 2:** Hierarchical Regression Predicting Child Outcomes From ΔERN amplitude, Asymmetry, and the interaction between ΔERN amplitude and Alpha Asymmetry.

	Social Inhibition				ADHD			
	*B*	*SE(B)*	Β	95% *CI*	*B*	*SE(B)*	β	95% CI
**Frontal Asymmetry**								
ΔERN at Cz	0.00	0.01	0.03	[-0.02, 0.02]	0.00	0.01	0.01	[-0.01, 0.01]
Asymmetry	3.67	1.91	0.43	[-0.33, 7.68]	0.98	1.71	0.13	[-2.62, 4.58]
ΔERN*Asymmetry	-0.03	0.15	-0.05	[-0.36, 0.29]	-0.25	0.13	-0.43	[-0.53, 0.02]
		*R*^*2*^ = 0.19				*R*^*2*^ = 0.19		
**Parietal Asymmetry**								
ΔERN at Cz	0.01	0.01	0.23	[-0.01, 0.02]	0.00	0.01	0.05	[-0.01, 0.02]
Asymmetry	-0.72	0.78	-0.22	[-2.35, 0.92]	-0.14	0.69	-0.05	[-1.59, 1.31]
ΔERN*Asymmetry	0.13*	0.05	0.52*	[0.03, 0.24]	-0.00	0.05	-0.02	[-0.12, 0.11]
		*R*^*2*^ = 0.33				*R*^*2*^ = 0.00		

*p < 0.05

Frontal asymmetry and ΔERN at Cz did not interact to predict social inhibition during preschool (Δ*R*^2^ = 0.00, β = -0.05, *p* = .83). In contrast, parietal asymmetry and ΔERN interacted to predict preschoolers’ social inhibition (Δ*R*^2^ = 0.26, β = .52, *p* = .02). Consistent with the recommendations of Aiken & West [52], we probed this interaction by recentering ΔERN at high (+1 *SD*) and low (-1 *SD*) values. This method allowed for a continuous probe of the interaction,eliminating the need for the creation of arbitrary groups that would result in a loss of statistical power. Probing the interaction in this manner revealed that when ΔERN was greater (more negative values), less left asymmetry was associated with greater social inhibition (β = -0.88, *p* = .02). In contrast, when ΔERN was smaller, asymmetry was unrelated to social inhibition (β = 0.28, *p* = .34).

The ΔERN at Cz did not interact with either frontal (Δ*R*^2^ = 0.18, β = -0.43, *p* = .07; Table 2) or parietal asymmetry (β = -0.02, *p* = .95) to predict ADHD symptoms. Similarly, no main effects were significant (all βs< 0.14, *p*s > .57).

## Discussion

Through the current study, we offered initial support for the Joint Subsystems Hypothesis early in life. Results indicated the presence of a link between a neural index of BIS (i.e., the ERN) and a neural index of BAS (i.e., hemispheric asymmetry), during the preschool years. This association was most robust at parietal electrode sites, distinguishing it from the pattern previously reported in adults [7]. We also provided evidence that early interactions between ERN and asymmetry have implications for children’s longitudinal outcomes, particularly in association with risk for anxiety problems (i.e., social inhibition).

Consistent with theoretical work that has posited such associations [6], [53], the present findings offer direct neural evidence that a system denoting activation–or approach—orientation antagonizes a system denoting inhibition orientation. To date, only one study has used neural indices to directly test the possibility that BIS and BAS act in opposition [7]. Our study replicates this work, to an extent, and offers initial evidence that this interactive association is present long before adulthood. Thus, it is possible that BIS and BAS are either innately associated or that associations between these systems are initiated prior to the preschool years. It should be noted, however, that the correlations between ERN and hemispheric asymmetry in the current work were smaller than those reported by Nash and colleagues, suggesting that associations may strengthen over time. Understanding when these associations emerge and how they develop over time will thus be an important area for future research.

It should also be noted that, when comparing the current work to previous or future research, that methods for calculating ERN amplitudes vary. In the current study, we used a difference wave calculation in order to isolate neural processes that are specific to error trials and not simply reflective of response processing that might be common to both correct and error trials. The use of difference waves is ideal for isolating neural processes in this way [50]. Nonetheless, readers should keep in mind that our difference wave reflects a difference between the error and correct trial conditions; it may not be appropriate to compare our study directly to work that defines ERN using only error-trial amplitudes. Although patterns in our data are similar when only error trials are examined, these two types of measures may not reflect exactly the same aspects of underlying neural processes. Similarly, the use of a developmental sample may prevent direct comparison with adult samples. For example, our sample of young children produced relatively large-voltage ERNs. While this may also be partially attributable to our use of a difference wave for calculating ERN, enhanced amplitudes in child, relative to adult, samples is not uncommon [54] and have been previously reported for ERN [24], [46]. Additionally, our results suggest an onset of the ERN, in some cases, that occurs prior to the participant response. In fact, it is not uncommon to observe an onset or peak for ERN prior to 0 ms, or the time of the participant’s response [24], [55], [56]. Previous work has shown a similar phenomenon, thought to result from the onset of responses occurring prior to the button press to which data are locked. As a reflection of this, it is common to score ERN during a window that ranges from shortly prior to the response to shortly after the response, as was the procedure followed here.

Our findings were also unique from previous research in that we observed associations between ERN and asymmetry primarily at parietal sites. Given that neural development is known to be protracted in frontal areas of the brain in particular [37], it makes some sense that associations between ERN and frontal asymmetry may be present but not fully developed by age 4. Indeed, these initial results may reflect an effect that is constrained to the early childhood years, when neural activity is more diffuse and inefficient as systems mature [57], [58]. However, to our knowledge, research with adults has not yet directly tested links between ERN and parietal asymmetry. It is therefore difficult to know whether our findings using measures of parietal asymmetry are unique to child populations or whether the same effects might be visible in adults. Although less is known about associations between temperament traits and parietal asymmetry than associations between temperament and frontal asymmetry, research with adults has suggested that greater right parietal asymmetry is associated with greater affective arousal [59] and social approach [60], which are notably reminiscent of BAS dimensions. In addition, it has been suggested that right posterior asymmetry is positively linked to anxiety problems [61] and negatively linked to ADHD [34]. Together, this evidence suggests that parietal asymmetry may denote BAS tendencies in young children. This possibility will be important to consider as the neural correlates of inhibition and activation systems continue to be delineated.

Our work also extends previous research on interactions between the ERN and asymmetry by testing their associations with ADHD and internalizing problems six months later. That ERN-asymmetry interactions were unrelated to ADHD symptoms was unexpected. It may be the case that BIS-BAS interactions are less important for ADHD symptoms than for internalizing behaviors in children. Indeed, neural markers of BIS and BAS are less frequently reported as predictors of symptoms of ADHD than symptoms of anxiety risk. In fact, to our knowledge, only a handful of studies have examined links between ADHD symptoms and hemispheric asymmetry in young children [34], [35], [62]. Results from this work are inconsistent, with some studies reporting increased activity in the right hemisphere for children with ADHD and some work reporting decreased activity in the right hemisphere for children with ADHD. One possible explanation for this variability may be that ADHD can be decomposed into constituent subtypes (i.e., inattentive vs. impulsive [63]) that may be more precisely associated with neural markers of risk. However, our results remain unchanged when analyses are conducted separately for symptoms of inattention and impulsivity. Another possibility is that scales such as that used here, intended to measure levels of early symptoms, are not ideal for measuring pre-clinical levels of risk. As few children would be expected to have diagnoses at age 4, our analyses were intended to assess symptoms that may denote enhanced risk for later disorder. A more proximal measure of risk constructs, such as a direct assessment of temperamental surgency [30], may provide a more sensitive test of this interaction. Additional research will be necessary to clarify links between ERN, hemispheric asymmetry, and putative risk for ADHD. Future work should also capitalize on the power of multi-trait, multi-method designs for characterizing child symptoms across a broader range of disorders. A reliance on one reporter may minimize symptoms that manifest primarily in other domains. Similarly, a reliance on one type of problem may ignore important manifestations of other relevant psychological disorders. This work, for example, may benefit in particular from the inclusion of teacher-reported symptoms of ADHD or other disorders (e.g., Oppositional Defiant Disorder) that occur outside of the purview of parents.

In contrast, we found that the combination of enhanced ERN and decreased left asymmetry predicted high levels of social inhibition, a known precursor to anxiety problems during childhood and adolescence [29], [64], six months later. This finding is consistent with the Joint Subsystems Hypothesis postulate that BAS activity mitigates BIS activity. For individuals who are prone to anxiety problems, BAS activity may offset propensities for BIS overarousal [6]. Our results suggest that under circumstances where BIS activity is not offset by BAS activity—that is, when both greater ERN and less left asymmetry are observed–risk for anxiety problems may be enhanced. That this pattern of results only emerged at parietal sites may indicate a greater relative importance of indices of arousal in the early years, as emotional [65], [66] and neural systems mature [37], [58] and links between systems become established.

Despite the contributions of the current study, this work is not without limitations. First, although our sample size was similar to that employed in previous work [7], our results were derived from a small sample of children. If the observed correlations are consistent with the true size of the effect in the population, our achieved power (α = 0.05) was between 0.58 and 0.71 for detecting true associations between ΔERN and asymmetry. Similarly, our achieved power was 0.84 for detecting a true interaction between ΔERN and asymmetry predicting social inhibition. Nonetheless, our power to detect small effects remains limited. Second, the current work was specifically designed to assess risk factors for psychopathology in a community sample. Neither counts of clinical symptoms nor diagnoses for children are available. As a result, the degree to which our results can be generalized to a clinical sample is unknown. In addition, although our study used a longitudinal design, it is unclear whether the associations reported here predict subsequent diagnoses and severity across childhood. These issues will be important to address in future research. Finally, we recognize that data from young children, particularly those in the toddler-preschool years, likely contain more artifacts than would data from adults. That is not to say that such data should be discounted, given the benefits of this type of work for understanding neural development. However, a strong grounding of empirical investigations in theory and additional replications of this work will be essential for drawing long-term conclusions.

Nevertheless, this study offers what is, to our knowledge, the first evidence that early neural markers of BIS and BAS interact during the preschool years. Similar to interactions in adults, BAS activity, indexed by hemispheric asymmetry, appears to serve as an antagonist of BIS arousal (i.e., ERN amplitude). We also found that interactions between ERN and asymmetry are associated with social inhibition, a marker of anxiety risk, during early childhood. Overall, this work replicates and extends the literature on the Joint Subsystems Hypothesis and neural correlates of early risk for mental illness in young children. In it, we take initial steps to further elucidate the nature of interactions among neurocognitive systems in ways that shape childhood outcomes. Specifically, this approach aids in the elucidation of mechanisms that may both enhance or buffer individual levels of risk for ADHD and anxiety problems.
